# Technology at the service of surgery in a new technique of autotransplantation by guided surgery: a case report

**DOI:** 10.1186/s12903-020-01095-6

**Published:** 2020-04-07

**Authors:** J. Mena-Álvarez, E. Riad-Deglow, N. Quispe-López, C. Rico-Romano, A. Zubizarreta-Macho

**Affiliations:** 1 Head Academic, Master Degree in Endodontics, Faculty of Health Sciences, Alfonso X el Sabio University, Madrid, Spain; 2 Associate professor, Master Degree in Implants, Faculty of Health Sciences, Alfonso X el Sabio University, Madrid, Spain; 3 Associate professor, Department of Endodontics, Faculty of Health Sciences, Alfonso X el Sabio University, Madrid, Spain

**Keywords:** Autotransplantation, Guided autotransplantation, Guided surgery, Surgical template, 3D dental replica

## Abstract

**Background:**

The aim of this case report was to use a surgical technique for autotransplantation of tooth using virtually planned 3D printed surgical templates for guided osteotomy preparation of the recipient of donor tooth.

**Case presentation:**

An 18-year-old male patient received autotransplantation of the right mandibular third molar to replace an included right second molar. This procedure was based on guided implant surgery methods by superimposition of DICOM files and 3D data sets of the jaws. In order to design a 3D-printed template with the aid of a fully digital workflow; the third molar was conserved in PRGF during the surgical procedure and the tooth socket was prepared with a template and the help of a 3D-printed donor tooth copy in order to prevent iatrogenic damage to the donor tooth. This template and replica were manufactured using 3D-printing techniques. The transplanted tooth was placed in infra-occlusion and fixed with a suture splint and root canal therapy was performed 15 days later. The intervention was be accomplished by performing preplanned virtual transplantations with guided osteotomies to ensure accurate donor tooth placement in the new recipient site. The 24 months follow-up showed physiological clinical and radiologic results compatible with healing periradicular tissues.

**Conclusions:**

This approach enables the planning and production of a 3D printed surgical template using the latest diagnostic methods and techniques of guided implant surgery. These accurate virtually predesigned surgical templates and printed analogues of the donor tooth could facilitate autotransplantation, ensuring an atraumatic surgical protocol.

## Background

Transplantation of teeth in dentistry is not a novel technique and has been used for many years. Nowadays, autotransplantation is almost exclusively used, and is defined as a clinical procedure where donor teeth are inserted into newly created alveoli in the same individual [1, 2].

Autotransplantation is recommended in teenagers who have not completed their growth [1, 3, 4] because it increases the chances of success. There are many reasons for tooth autotransplantation, but the most common indication is the natural replacement of a tooth as a result of complicated dental fracture, deep caries or failure in endodontic treatment. In addition, tooth transplantation may be employed to treat patients with congenital tooth absence, pre-existing impacted or ectopic teeth and spaces [4–6]. Autotransplantation is recommended in patients with orthodontic indications, which requires extraction of their permanent teeth when potential donors are available. In these cases the extracted teeth, commonly first premolars, can be used to replace missing teeth, usually the anterior teeth because of their favourable morphology, size and single root canal. Some restorative techniques can be used to modify premolars and make them simulate incisor teeth [7–9].

The average success rate for autotransplantations varies between 79 and 100%. This is due to the variable follow-up time and the different number of interventions performed in the analyzed studies [1–4, 10, 11]. The outcome of autotransplantation may be influenced by complications caused by documented risk factors, such a extraction need and failure, hypermobility, pulp necrosis, pulp obliteration, root resorption) [4], although they can present promising results and be successful. The wound healing and biological principles are similar to avulsed teeth after replantation because of trauma. Consequently, if it exists traumatic press-fit placement in the recipient alveolus or mechanical injuries during extraction, in addition to biochemical factors because of prolonged extraalveolar duration may cause damage to the periodontal ligament, leading to progressive root resorption and treatment failure. Others factors like root morphology and development or inadequate preparation of the recipient alveolus, appear to influence a negative outcome, causing pulp necrosis and root resorption, ankylosis and failure [4, 12, 13]. Restorations with dental implants and prosthetic bridges (fixed treatment options) cannot allow for natural functional and esthetic rehabilitation like dental transplants. Moreover, teeth preparation for prosthetic restorations, may incur in pulp damage that may require root canal treatment of the abutment teeth [2, 4]. Conversely, autotransplantation will provide for proprioception during function [2, 4]. In contrast to dental implants, autotransplantation provides the advantage of the possibility of carrying out orthodontic treatment of teeth, if required [1, 2].

Platelets concentrates derived from the patient’s whole blood contains growth factors. Studies have shown that specific growth factors (GF) which are contained in the α-granules of platelets, may favour the bone regeneration in oral bone defects. These GFs have anti-inflammatory and healing properties that help the aesthetic results. Furthermore the use of platelets concentrates may generate positive facts like shortened duration of treatment and reduction of postoperative symptoms [14, 15].

Preoperative cone-beam computed tomography (CBCT) planning and the use of 3D-printed replicas of the donor teeth to prepare the artificial socket are specifically described in autotransplantation [16–19]. A navigation system, working on CBCT data and a computer assisted software, able to guide the high-speed hand piece (and bur) to the exact position may present a challenging approach. This kind of technology has already been used for dental implantology for several years using the standard drill and templates [20, 21].

The aim of this paper is to present a new surgical technique using virtually planned 3D for guided osteotomy preparation of recipient site in autotransplantation and safe placement of donor teeth by means of a 3D printed replica and surgical templates. By the implementation of innovative methods, this approach could ensure an atraumatic and precise autotransplantation of teeth.

## Case presentation

**Fig. 1:**
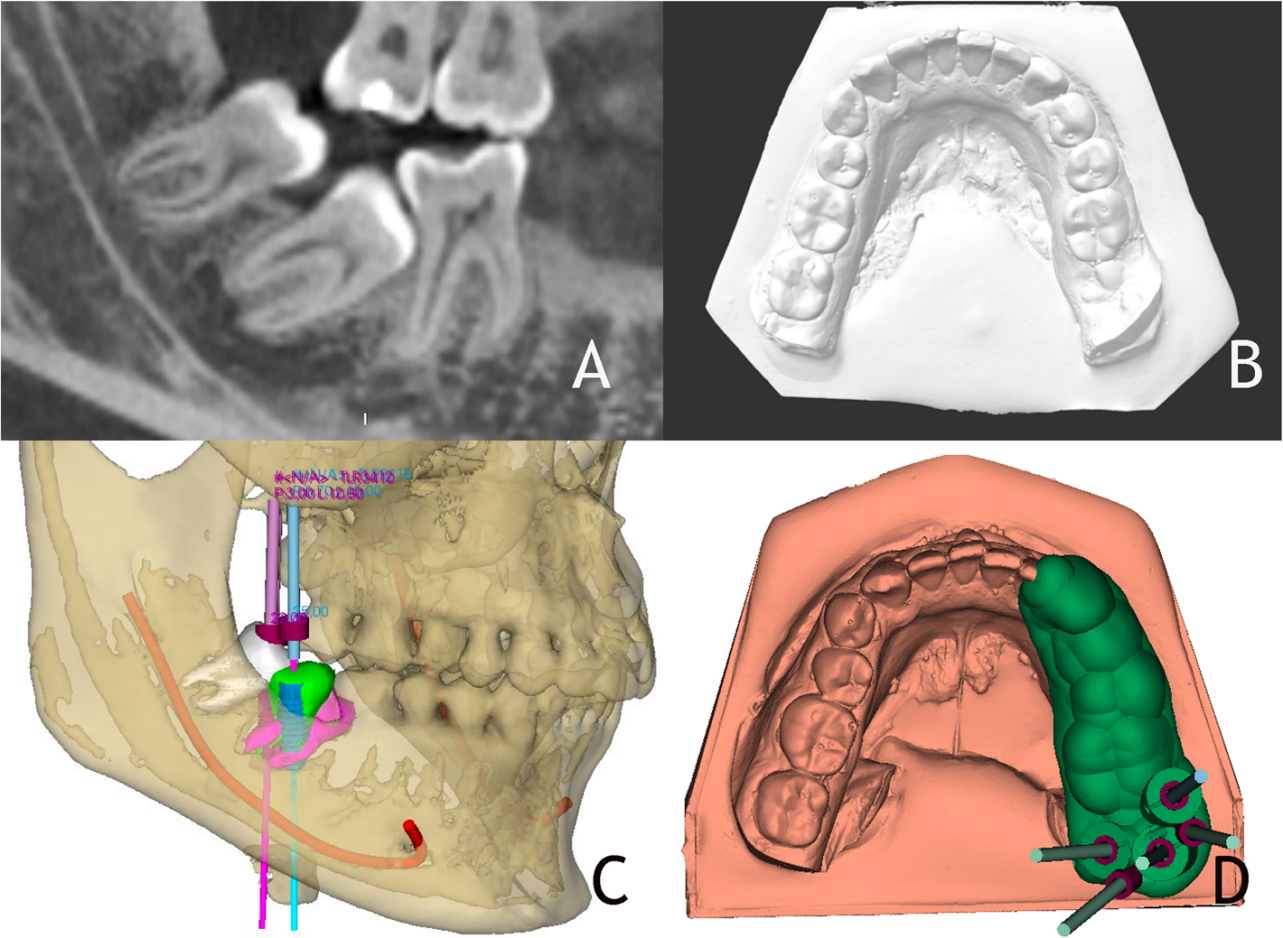
**a** An 18-year-old male patient was referred with the mandibular third and second molar included. Sagital view in CBCT (White Fox) reveals the possibility of performing an autotransplantation. **b** Dental models scanned by scanner extraoral (EVO). **c** Virtual autotransplantation (green tooth) of tooth #48 (white tooth) into the place of tooth #47 (pink tooth), and drilling planning procedure. **d** Surgical template and models exported as STL files and sent to a 3D printer for fabrication

An 18 year-old male patient was referred for evaluation of the third and second mandibular molar included. Radiographic examination confirmed the diagnosis. A complementary radiographic examination was performed to ensure accurate treatment planning with CBCT (White Fox, Acteon Medico-Dental Iberica S.A.U, Satelec, Merignac, France), (Fig. 1a) with exposure parameters of 105.0 kVp, 8.0 mA, 7.20 s and a field of view of 6 × 6 mm. The dental models were scanned by an extraoral scanner (EVO, Ceratomic, Protechno, Gerona, Spain) and segmented stereolithography (STL) files were analyzed. (Fig. 1b).

After making an interdisciplinary consultation, the orthodontic treatment was not considered a treatment option for the patient. Therefore, autotransplantation of the right mandibular third molar into the place of the right mandibular second molar was planned as the most appropriate treatment plan. The reason to perform a double extraction was that the patient had already undergone orthodontic treatment when he was 8 years old and afterwards he used a multibracket device when he was 13 years old, which ended in a refusal to undergo a new orthodontic treatment. We knew that the best treatment would have been orthodontics with extraction of the third mandibular molar and second mandibular molar alignment by using miniscrews or mini-implants but the patient refused such treatment and that is why we gave him a second option with an autotransplant of the third molar.

Both digital files obtained by CBCT and extraoral scanner ((Digital Imaging and Medical Communications Files (DICOM) and Standard Triangle Language (STL)) were imported into surgical planning software designed for guided implant surgery (NemoScan, Explora 3D lab, Nemotec S.L., Arroyomolinos, Madrid, Spain) to digitally position the right mandibular third molar. In carrying out autotransplantation in the present case, CBCT images and 3D simulation were particularly useful for surgical simulation.

Similarly to planning using virtual guides of dental implants, the rotation of donor tooth and correct angulation was decided; the accurate positioning of the donor tooth was defined with the aid of STL files of donor tooth and with five surgical pins. The alveoli of the two radicular processes of the right mandibular third molar were created by means of a 3D printed tooth-supported surgical template with five drilling sleeves of ϕ 2.5 mm. The exact 3D position was selected based on its position with respect to the antagonist teeth in the upper arch must be taken into account to ensure functional restoration after surgical intervention (Fig. 1c). This was virtually planned using rotatory surgical instruments. The use of technology planning through CBCT was very useful in this case despite having an alveolus partially created since the axis of extracted tooth is different from the autotransplanted tooth positioned.

Technical STL files of surgical burs and dental implants (Guided Implant Surgery Biohorizons; Biohorizons S. L, Birmingham, USA) were visualized and superimposed onto the osteotomy plan to facilitate final treatment planning. Afterwards, these files were imported into planning software to obtain accurate osteotomies. Once the position and dimension of donor tooth had been virtually designed, a surgical template for guided osteotomy was developed within the software. This template was virtually designed to ensure precise positioning throughout surgical intervention. As a final part of process, the surgical template was exported as STL files and sent to a 3D printer for production (Explora 3D Lab, Nemotec S. L, Arroyomolinos, Madrid, Spain) (Fig. 1d).

**Fig. 2:**
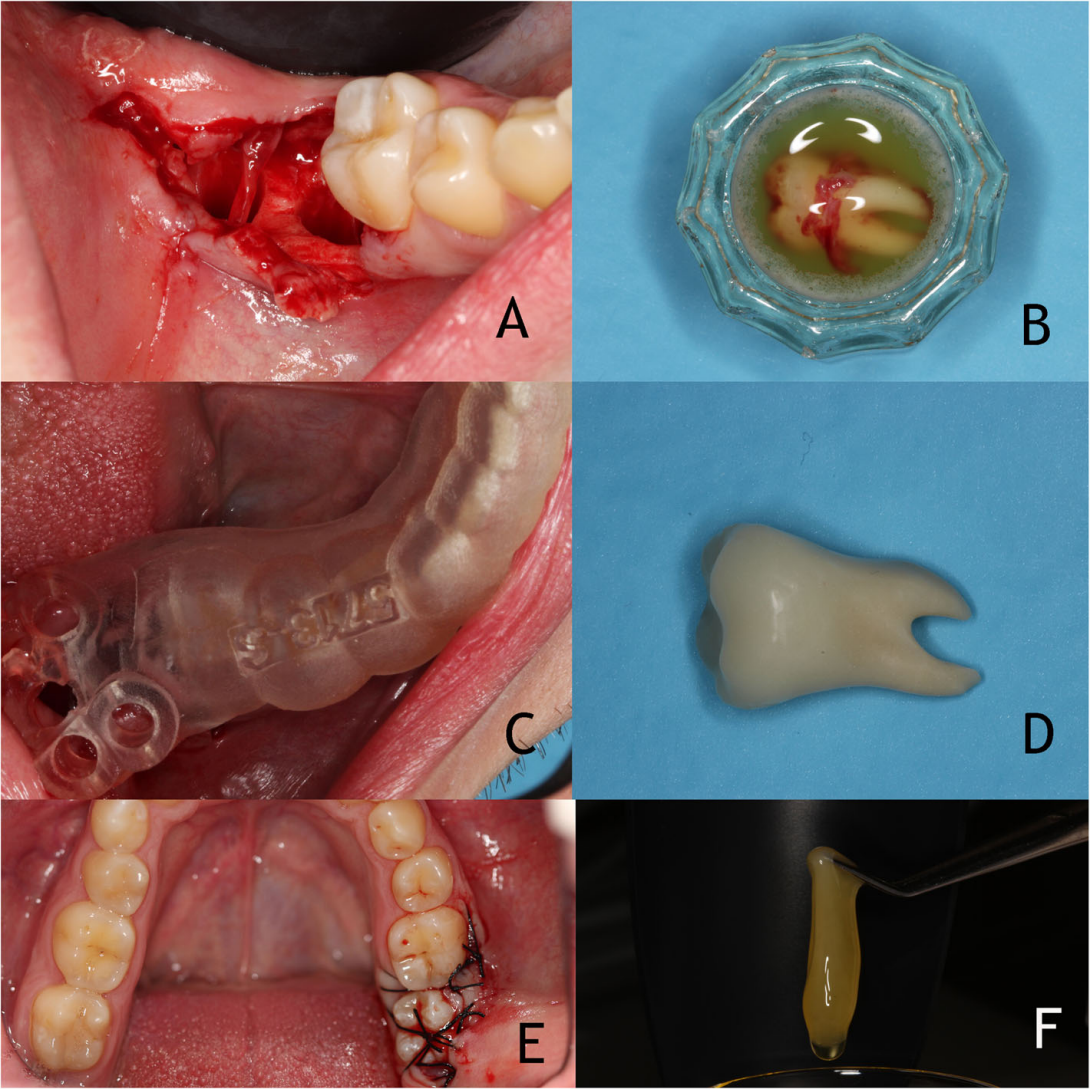
**a** Atraumatic extraction of right mandibular third molar without any bone removal. **b** Third molar conserved in autologous plasma rich in growth factors (BTI Biotechnology) while the extraction of the second molar and the surgery procedure. **c** Confirmation of stability of the 3D printed surgical template before starting the drilling guided procedure. **d** 3D tooth replica used to verify the final preparation of the recipient site. **e** Tooth positioned in new alveolus and sutured. **f** Activated PRGF ready to be applied around the autotransplanted tooth

Surgery was performed under local anaesthesia using articaine 2% and 1:100000 epinephrine (Artinibsa, Inibsa S.A., Lliça de Vall, Barcelona, Spain). The right mandibular third molar was extracted atraumatically (Fig. 2a) without any bone removal with forceps and it was preserved in autologous plasma rich in growth factors (PRGF) (BTI Biotechnology Institute, Vitoria, Spain) while the extraction of the second molar and the surgery procedure were being carried out (Fig. 2b). The extraoral time was measured and the root surface was not manipulated to enhance PDL fiber attachment. The stability of the 3D printed surgical template was confirmed, before starting the drilling guided procedure (Fig. 2c). The surgical template with five drilling of Ø 2.5 mm was positioned. All drills were 2.5 mm diameter and the first drill was introduced at 15 mm, the second and the third drill at 12 mm and the fourth and fifth drill at 7.5 mm.

A 3D tooth replica was inserted to verify the final preparation of the recipient site (Fig. 2d) but the adaptation was not perfect and, after the position and stability of the dental replica were confirmed, the autotransplanted tooth was inserted and mesially and distally stabilized with sutures across the occlusal plane (Fig. 2e). The total extraoral time was 17 min as a result of guided surgery. Activated PRGF was then applied around the autotransplanted tooth (Fig. 2f). Occlusal contacts were gently removed to keep the transplant from postoperative traumatic occlusal forces. An antibiotic (amoxicillin 750 mg every 8 h), an analgesic (ibuprofen 600 mg) and an antibacterial chlorhexidine gluconate rinse (0.20%) were prescribed for 1 week. The patient was also advised to eat a soft diet for the first few days. The healing process was uneventful and there were no postoperative complications.

**Fig. 3:**
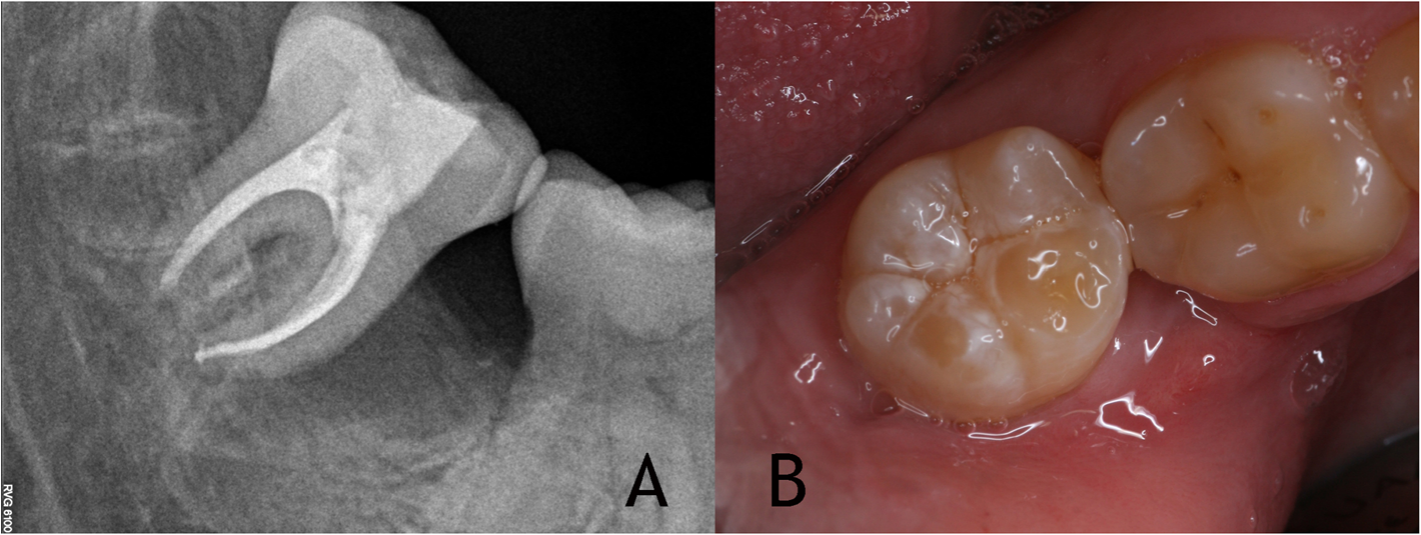
**a** Root canal treatment performed 15 days after autotransplantation. **b** Clinical image before carrying out the endodontic treatment

Two weeks later, the root canal treatment was performed. A rubber dam (Hygenic® dental dam, Coltene Whaledent Grouppe, Altstätten, Switzerland) was used to achieve total isolation and the working length was determined with an electronic apex locator (Raypex 6®, VDW, Munich, Germany). Preparation of the root canal was performed using Protaper Next® rotary instruments (DentsplySirona Endodontics, Ballaigues, Switzerland). Final preparation of the canals was done with X2 in the mesial canals and X3 in the distal canal. Afterwards, an apical calibration was done to determine the real canal diameter. The smear layer was removed with 5.25% NaOCl and 17% EDTA and activated with Endoactivator® (Dentsply Sirona Endodontics, Ballaigues, Switzerland). After the completion of cleaning and shaping of root canal the treatment was completed using warm vertical condensation technique (Elements®, Sybron Endo, Orange, USA) for obturation of the canals and an epoxy resin cement (AH Plus®, Dentsply DeTrey, Konstanz, Germany) (Fig. 3a). The tooth crown was permanently restored with resin.

The patient underwent clinical and radiographic follow-ups after 1 day (post- operative), 1 week (suture removal), 2 weeks (root canal treatment), 1 month (clinical examination) and 6, 12, 18 and 24 months (clinical/radiographic examinations). The patient was asymptomatic during the post-operative period (Fig. 3b).

**Fig. 4:**
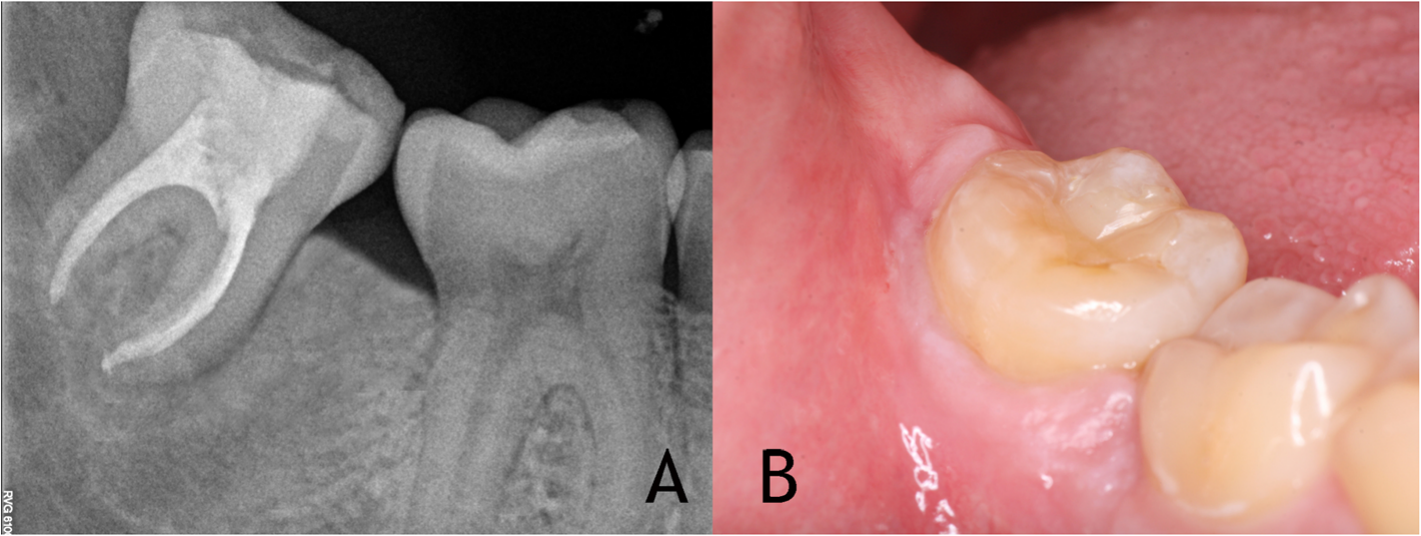
**a** The 24 months radiographic follow-up showing normal bone regeneration in the site of previous third molar and normal periodontal ligament with no signs of pathology of root resorption. **b** Asymptomatic patient and transplanted tooth is functional with no signs of marginal periodontal pathosis

**Fig. 5:**
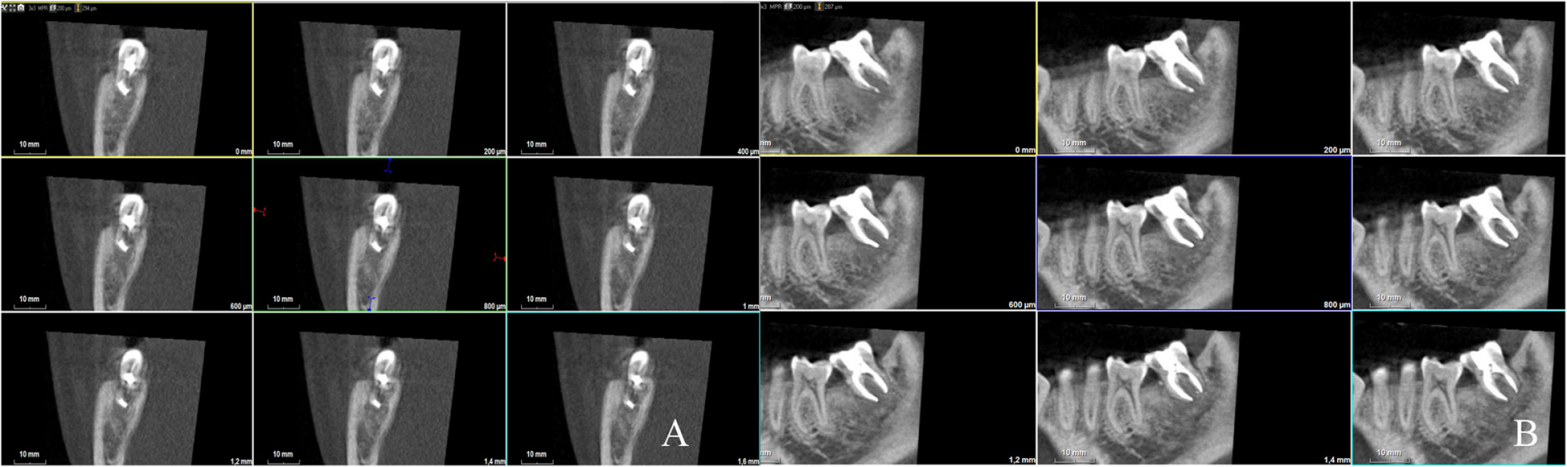
**a** Axial view in CBCT (White Fox) 24 months follow-up. **b** Sagital view in CBCT (White Fox) 24 months follow-up reveals bone regeneration

After 24 months, clinical and radiographic examinations revealed satisfying results, with no signs and symptoms. The patient has no symptoms and the transplanted tooth is functional with no signs of marginal periodontal pathology. Radiographies and CBCT showed bone regeneration in the site of previous third molar, normal periodontal ligament with no signs of pathology of root resorption (Figs. 4a, b, 5a and b).

## Discussion and conclusions

Tooth transplantation can be categorized into several types: autogenous, homogenous and heterogeneous [22, 23]. Autogenous tooth transplantation or autotransplantation is a tooth surgical transplantation from its original site in the mouth to another site in the same person [22].

Transplanted teeth with complete root formation have a 15% rate of pulpal healing, compared with 96% for transplanted teeth with incomplete root formation [24]. A systematic review has provided evidence of the outcome of autotransplanted teeth with complete root formation [3]. Ankylosis, root resorption and rates of failure have been infrequently observed in autotransplanted teeth with fully developed roots. Although autotransplantation is possible for teeth with completed root formation, endodontic treatment is as preferrably performed before transplantation because pulpal healing and revascularization are less likely to occur [25]. Thus, the prognosis improves preventing inflammatory procedures that could endanger the success of the treatment [26]. Root canal treatment must be performed 7 to 14 days after transplantation; otherwise, the necrotic pulp may result in inflammatory resorption. According to the classification of Andreasen et al. [24], in our case the tooth development was at stage 6 (complete root formation, apical foramen half closed) and because the healing of the pulp was not expected, root canal treatment was performed 2 weeks after transplantation in one session without calcium hydroxide although the literature reflects treatment in 2 sessions with calcium hydroxide.

The vitality of the favorable periodontal ligament (PDL) attached to the transplanted tooth [25] is the most important factor for the success of autogenous tooth transplantation and this viability is reduced if the extraoral dry time is long [13]. In this case, the tooth was held gently and conserved in plasma rich in growth factors. Vavouraki et al. reported that the addition of PRGF to the PDL cells culture produced statistically increased proliferation of PDL cell [27]. An extraoral time less than 20 min is associated with a positive effect on tooth survival [13, 28, 29]. In this case report, the total extraoral time was only 17 min, and the root surface was not manipulated to enhance PDL fiber attachment and prevent ankylosis. In this case, the previous measurements performed using CBCT imaging, the elaboration of one prototyping model and surgical templates designed for the preparation of the recipient site were essential to minimize the extraoral time [30, 31].

Strbac et al. in 2016^18^ reported a case clinic based in similar technology in avulsion of permanent maxillary incisors. The development of software and fabrication of surgical templates by implementing STL files of dental models, generating precise 3D printed surgical templates, were used for the present case to deploy a planning technique, reducing time extraoral and improving the success of the autotransplantation of third molar. The use of a three-dimensional printed analogue of the donor tooth from a CBCT scan of the tooth is described in the literature [16, 17, 19, 32]. In this case, a 3D dental replica was used in order to minimize the extra-oral time and frequency of trial insertions of the donor tooth into the recipient socket which was elaborated by the surgical templates and the surgical drill of implants and the adaptation of de socket was virtually perfect.

With the use of PRGF, patients experience minimal pain postoperative, reduced infection rates and overall improved healing, making future root canal treatment unnecessary for the transplanted teeth if the apex is immature but in this case, the apex is mature and endodontic treatment was required [14, 15, 33, 34]. There are some publications with PRP [33] and few [15] or none with PRG-F and auto-transplants. Anitua et al. [15] already kept the tooth in PRGF during the surgical process, also to using it as soft tissue healing. The benefit of soft tissue is evident, but it is not known in bone healing. After the improvement in soft tissue healing of our case report we could say that the use of PRGF enhanced the healing and recovery of the tooth autotransplantation technique. It is necessary to carry out more cases to be able to know the advantages of the PRGF in autotransplants.

Excessive time or rigid splinting of the transplanted tooth will adversely affect its healing outcome. Most reports advise flexible splinting for 7 to 10 days with sutures placed through the mucosa and over the occlusal surface of the crown because this permits some functional movement of the transplant and stimulates periodontal ligament cellular activity and bone repair [35, 36]. In this case, the suture was retirated at 7 days and manufactured as described above.

This alternative option of treatment should be presented to patients that are in need of extraction of a posterior tooth (first or second molar) and possess impacted/non-functional third molars anatomically viable for this technique.

To conclude, we can say that it is possible to use diagnostic methods and techniques of guided implant surgery in endodontics, enabling the planning and production of 3D printed surgical templates. The 3D printed surgical template and dental replica obtained from a therapeutic planning based on CBCT and STL files, can make a conservative, accurate and safe osteotomy, which affect the stability and prognosis of the autotransplanted teeth. More cases studies with PRGF would be necessary to know the advantages of this technique, besides carrying out a study where all the variables involved could be analyzed.

## Data Availability

All data generated or analysed which related this case report are included in this published article.

## Abbreviations

CBCT: Cone-beam computed tomography
DICOM: Digital Imaging and Communications in Medicine files
EDTA: Ethylenediaminetetraacetic acid
GF: Growth factors
NaOCl: Sodium hypochlorite
STL: Standard Triangle Language
PDL: Periodontal ligament
PRGF: Plasma rich in growth factors
